# A loss of telocytes accompanies fibrosis of multiple organs in systemic sclerosis

**DOI:** 10.1111/jcmm.12228

**Published:** 2014-01-28

**Authors:** Mirko Manetti, Irene Rosa, Luca Messerini, Serena Guiducci, Marco Matucci-Cerinic, Lidia Ibba-Manneschi

**Affiliations:** a Department of Experimental and Clinical Medicine, Section of Anatomy and Histology, University of FlorenceFlorence, Italy; b Department of Experimental and Clinical Medicine, Section of Surgery, Histopathology and Molecular Pathology, University of FlorenceFlorence, Italy; c Department of Experimental and Clinical Medicine, Section of Internal Medicine, Rheumatology Unit, University of FlorenceFlorence, Italy

**Keywords:** telocytes, systemic sclerosis, scleroderma, fibrosis, gastric wall, myocardium, lung, CD34, immunohistochemistry

## Abstract

Systemic sclerosis (SSc) is a complex connective tissue disease characterized by fibrosis of the skin and various internal organs. In SSc, telocytes, a peculiar type of stromal (interstitial) cells, display severe ultrastructural damages and are progressively lost from the clinically affected skin. The aim of the present work was to investigate the presence and distribution of telocytes in the internal organs of SSc patients. Archival paraffin-embedded samples of gastric wall, myocardium and lung from SSc patients and controls were collected. Tissue sections were stained with Masson's trichrome to detect fibrosis. Telocytes were studied on tissue sections subjected to CD34 immunostaining. CD34/CD31 double immunofluorescence was performed to unequivocally differentiate telocytes (CD34-positive/CD31-negative) from vascular endothelial cells (CD34-positive/CD31-positive). Few telocytes entrapped in the fibrotic extracellular matrix were found in the muscularis mucosae and submucosa of SSc gastric wall. In the muscle layers and myenteric plexus, the network of telocytes was discontinuous or even completely absent around smooth muscle cells and ganglia. Telocytes were almost completely absent in fibrotic areas of SSc myocardium. In SSc fibrotic lung, few or no telocytes were observed in the thickened alveolar septa, around blood vessels and in the interstitial space surrounding terminal and respiratory bronchioles. In SSc, the loss of telocytes is not restricted to the skin, but it is a widespread process affecting multiple organs targeted by the fibrotic process. As telocytes are believed to be key players in the regulation of tissue/organ homoeostasis, our data suggest that telocyte loss might have important pathophysiological implications in SSc.

## Introduction

Systemic sclerosis (SSc), or scleroderma, is a complex connective tissue disease characterized by widespread fibrosis involving the skin and multiple internal organs, especially lungs, heart and gastrointestinal tract 1,2. The progressive fibrotic process disrupts the physiological structure of the affected tissues and frequently leads to significant organ dysfunction. Therefore, visceral organ fibrosis is responsible for significant morbidity and is also a major cause of death in patients with SSc 1,2. The hallmark pulmonary histopathological lesion is non-specific interstitial pneumonia characterized by cellular inflammation and fairly uniform interstitial fibrosis, which manifests as progressive thickening of the alveolar septa, ultimately resulting in alveolar airspace obliteration with consequent restrictive lung disease 3,4. Cardiac manifestations include myocardial fibrosis, hypertrophy and disorders of the coronary and conduction systems that can lead to severe clinical complications such as congestive heart failure, arrhythmias and sudden cardiac death 5,6. Gastrointestinal involvement is commonly found in up to 90% of SSc patients 7. Although the distal oesophagus is virtually always affected, the entire gastrointestinal tract can be involved, showing fibrotic lesions in the muscularis mucosae, submucosa and muscle layers together with smooth muscle cell atrophy 8–3^10^. Clinically, gastrointestinal tract dysmotility is a major visceral manifestation, ranging from an asymptomatic form to severe paresis 7.

In SSc, fibroblasts are considered the principal effector cells 11, and their chronic dysregulation may result in resistance to proapoptotic stimuli, increased transition to myofibroblasts and excessive production and deposition of collagens and other extracellular matrix components 11,12. Moreover, vascular endothelial cells and pericytes and cells of both the innate and adaptive immune systems are also dysregulated in SSc and may contribute to fibroblast activation and fibrosis 11–3^13^.

Besides fibroblasts, there is evidence that other stromal (interstitial) cell types may be implicated in the pathophysiology of SSc. Telocytes are a peculiar type of stromal cells recently identified in a variety of tissues and organs of humans and laboratory mammals 14–3^17^. By their extremely long cytoplasmic processes, named telopodes, telocytes form a three-dimensional network that may function as a scaffold to define the correct organization of tissues/organs during pre-natal life or their repair/renewal in post-natal life 16–3^18^. According to their specific locations within different organs, telocytes may participate in intercellular signalling, either by cell-to-cell contacts or by secreting paracrine signalling molecules, immune surveillance and tissue regeneration by forming tandem cell structures with stem-cell niches 16–3^25^. In the gastrointestinal tract, telocytes have also been proposed to participate in the regulation of neurotransmission by spreading the slow waves generated by the pacemaker interstitial cells of Cajal (ICC) 16,26.

Recently, we have shown that telocytes display severe ultrastructural damages and are progressively lost from the clinically affected skin of SSc patients, suggesting that telocyte loss might contribute to the altered three-dimensional organization of the extracellular matrix and reduce the control of fibroblast/myofibroblast activity, thus favouring chronic fibrosis 27.

As SSc is a multisystem fibrotic disorder 1,2, the aim of the present study was to investigate the presence and distribution of telocytes in the internal organs of SSc patients.

## Materials and methods

### Biopsy specimens and histochemistry

Paraffin-embedded samples of gastric wall, LV myocardium and lung from SSc patients (*n* = 5) and controls (*n* = 7) were collected at the archives of the Section of Surgery, Histopathology and Molecular Pathology and the Section of Anatomy and Histology, Department of Experimental and Clinical Medicine, University of Florence. Biopsy specimens from SSc patients were selected by reviewing the pathological records using the keywords ‘systemic sclerosis’ and ‘scleroderma’. Cases were included in the analysis if the review of the medical records identified clinical features that satisfied the criteria of LeRoy *et al*. 28 for a diagnosis of SSc. Pulmonary fibrosis and heart failure were the main causes of death recorded. As healthy control tissues, biopsy samples were obtained from autopsies or from patients who underwent surgery because of neoplastic pathologies. Specimens were taken at least 8 cm from the margins of the tumour. We carefully selected healthy specimens that appeared to have no inflammatory or neoplastic infiltration according to histopathological examination. All specimens had been fixed in 10% buffered formalin, dehydrated in a graded ethanol series and xylene, and embedded in paraffin. Sections were cut (5 μm thick) using a Leica RM2255 rotary microtome (Leica Microsystems, Mannheim, Germany), deparaffinized and stained with haematoxylin-eosin for routine histology and with Masson's trichrome with aniline blue (catalogue no. 04-010802; Bio-Optica, Milan, Italy) to detect fibrosis. Tissue sections were observed under a Leica DM4000 B microscope (Leica Microsystems), and transmitted light images were captured by using a Leica DFC310 FX 1.4-megapixel digital colour camera equipped with the Leica software application suite LAS V3.8 (Leica Microsystems).

### Immunohistochemistry

Immunohistochemistry was performed by using an indirect immunoperoxidase method. After deparaffinization, tissue sections (5 μm thick) were boiled for 10 min. in sodium citrate buffer (10 mM, pH 6.0) for antigen retrieval and treated with 3% H_2_O_2_ in methanol for 15 min. at room temperature to block endogenous peroxidase activity. Sections were then washed and incubated with Ultra V block (UltraVision Large Volume Detection System Anti-Polyvalent, HRP, catalogue no. TP-125-HL; LabVision, Fremont, CA, USA) for 10 min. at room temperature according to the manufacturer's protocol. After blocking non-specific site binding, slides were incubated overnight at 4°C with mouse monoclonal anti-human CD34 antibody (1:50 dilution; clone QBEnd-10, catalogue no. M7165; Dako, Glostrup, Denmark) diluted in 1% bovine serum albumin (BSA) in PBS. After washing in PBS, tissue sections were incubated with biotinylated secondary antibodies (UltraVision Large Volume Detection System Anti-Polyvalent, HRP, LabVision) for 10 min. at room temperature. Subsequently, the slides were washed in PBS and incubated with streptavidin peroxidase (UltraVision Large Volume Detection System Anti-Polyvalent, HRP, LabVision) for 10 min. at room temperature. Immunoreactivity was developed by using 3-amino-9-ethylcarbazole (AEC kit, catalogue no. TA-125-SA; LabVision) as chromogen. Sections were finally counterstained with haematoxylin, washed and mounted in an aqueous mounting medium (VectaMount™ AQ, Vector Laboratories, Burlingame, CA, USA) and observed under a Leica DM4000 B microscope equipped with fully automated transmitted light axes (Leica Microsystems). Sections not exposed to primary antibodies or incubated with isotype-matched and concentration-matched non-immune mouse IgG (Sigma-Aldrich, St. Louis, MO, USA) were included as negative controls for antibody specificity.

### Double immunofluorescence staining

Paraffin-embedded tissue sections (5 μm thick) were deparaffinized and boiled for 10 min. in sodium citrate buffer (10 mM, pH 6.0). Sections were washed in PBS, incubated in 2 mg/ml glycine for 10 min. to quench autofluorescence caused by free aldehydes and then blocked for 1 hr at room temperature with 1% BSA in PBS. For double immunofluorescence staining, the sections were incubated overnight at 4°C with a mixture of mouse monoclonal anti-human CD34 (1:50 dilution; Dako) and rabbit polyclonal anti-human CD31/platelet-endothelial cell adhesion molecule-1 (PECAM-1) (1:50 dilution; catalogue no. ab28364; Abcam, Cambridge, United Kingdom) antibodies diluted in PBS with 1% BSA. The day after, the slides were washed three times in PBS and incubated for 45 min. at room temperature in the dark with a mixture of Alexa Fluor-488-conjugated goat antimouse IgG and Rhodamine Red-X-conjugated goat anti-rabbit IgG (Invitrogen, San Diego, CA, USA) diluted 1:200 in PBS with 1% BSA. Irrelevant isotype-matched and concentration-matched mouse and rabbit IgG (Sigma-Aldrich) were used as negative controls. Cross-reactivity of secondary antibodies was tested in control experiments in which primary antibodies were omitted. Nuclei were counterstained with 4′,6-diamidino-2-phenylindole (DAPI; Chemicon International, Temecula, CA, USA). Tissue sections were then mounted with an antifade aqueous mounting medium (Biomeda Gel Mount, Electron Microscopy Sciences, Foster City, CA, USA) and examined with a Leica DM4000 B microscope equipped with fully automated fluorescence axes (Leica Microsystems). Fluorescence images were captured with a Leica DFC310 FX 1.4-megapixel digital colour camera equipped with the Leica software application suite LAS V3.8 (Leica Microsystems).

### Quantitative analysis

Quantitative analysis of telocytes was performed on tissue sections double-immunostained with the mouse monoclonal anti-CD34 antibody and the rabbit polyclonal anti-CD31/PECAM-1 antibody and counterstained with DAPI for nuclei. CD34-positive/CD31-negative telocytes were counted in 8 randomly chosen microscopic high-power fields (40× original magnification) per sample. Only the cells with well-defined nuclei were counted. Counting was performed by two independent observers who were blinded with regard to the sample classification. The final result was the mean of the two different observations for each sample. Data are represented as mean ± SD and were analysed by using the Student's *t*-test for independent samples. *P* < 0.05 was considered statistically significant.

## Results

**Fig 1:**
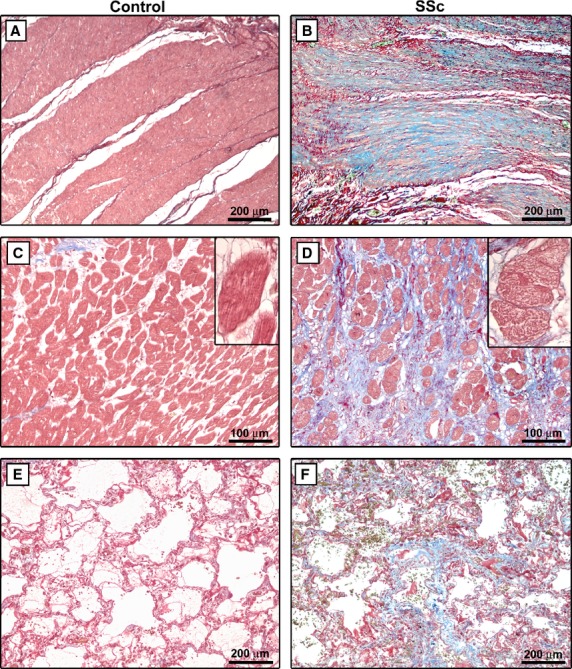
Masson's trichrome-stained sections of gastric wall (A and B), LV myocardium (C and D) and lung (E and F) from controls and patients with systemic sclerosis (SSc). (B) Muscle layers of SSc gastric wall display accumulation of dense collagen bundles surrounding atrophic smooth muscle cells. (D) In SSc myocardium, cardiomyocytes are embedded in fibrous tissue. *Inset*: At higher magnification view, SSc cardiomyocytes display hypertrophic changes and diffuse cytoplasmic vacuolization. (F) Lung sections from SSc patients display the typical features of non-specific interstitial pneumonia with diffuse cellular inflammation and collagen deposition resulting in thickening of alveolar septa and severe distortion of the pulmonary parenchyma structure. All tissue sections are stained with Masson's trichrome with aniline blue (red colour: cytoplasm; blue colour: collagen). Scale bars are indicated in each panel.

The histopathological examination of Masson's trichrome-stained sections of gastric wall, LV myocardium and lung showed the typical fibrotic changes of SSc as compared with control samples (Fig. 1A–F). A generalized fibrosis affected all layers of the gastric wall, with the most severe changes being observed in the muscularis mucosae, submucosa and muscularis propria. In particular, muscle layers displayed dense collagen bundles surrounding atrophic smooth muscle cells (Fig. 1B). As previously reported 8,9, the involvement of the muscle layers showed a patchy distribution, with fibrotic areas close to healthy-looking areas in the same tissue section. Wide areas of interstitial fibrosis and hypertrophy of cardiomyocytes were observed in SSc myocardium (Fig. 1D and inset). Lung sections from SSc patients displayed the typical features of non-specific interstitial pneumonia with both diffuse cellular inflammation and collagen deposition resulting in thickening of alveolar septa and severe distortion of the pulmonary parenchyma structure (Fig. 1F).

According to previously published studies 16,18,25–3^27^,^29^–3^33^, telocytes were identified by light microscopy using CD34 immunostaining. The distribution of telocytes in control specimens was consistent with that reported in the literature 26,29,31–3^33^.

**Fig 2:**
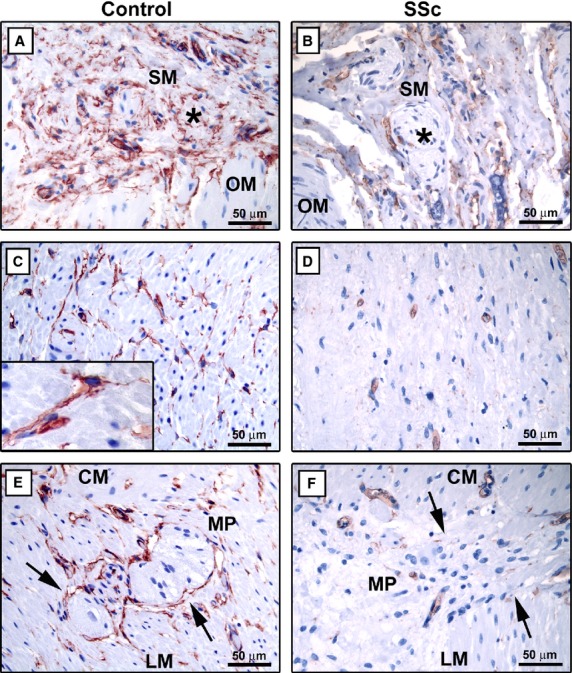
Gastric wall specimens from controls (A, C and E) and patients with systemic sclerosis (SSc) (B, D and F). (A–F) CD34 immunoperoxidase labelling with haematoxylin counterstain. (A and B) Submucosa. (A) In control gastric wall submucosa, telocytes form a network surrounding vessels and ganglia (*asterisk*) and encircle the submucosal border of the muscularis propria. (B) In the submucosa of SSc gastric wall, very few telocytes entrapped in the fibrotic extracellular matrix are observed. Note the absence of telocytes around submucosal ganglia (*asterisk*). (C and D) Muscle layers. (C) In the muscularis propria of control gastric wall, numerous telocytes surround smooth muscle bundles and cells. *Inset*: At higher magnification view, telocytes display a slender nucleated body and very long varicose processes located in the interstitium between smooth muscle cells. (D) Telocytes are very few or absent in muscle layers of SSc gastric wall. (E and F) Myenteric plexus. (E) In control specimens, telocytes form a network enveloping myenteric plexus ganglia (*arrows*) and are present along the nerve strands of the interganglionic region. (F) Note the almost complete absence of telocytes around myenteric plexus ganglia (*arrows*) of SSc gastric wall. SM: submucosa; OM: oblique muscle layer; CM: circular muscle layer; LM: longitudinal muscle layer; MP: myenteric plexus. Scale bars are indicated in each panel.

**Fig 3:**
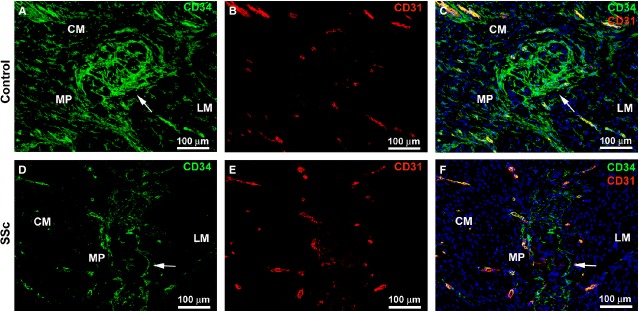
Gastric wall specimens from controls (A–C) and patients with systemic sclerosis (SSc) (D–F). (A–F) Double immunofluorescence labelling for CD34 (green) and CD31 (red) with DAPI (blue) counterstain for nuclei. Telocytes are CD34-positive and CD31-negative, while vascular endothelial cells are CD34/CD31-double-positive. (A–C) Control gastric wall. Telocytes form a network around smooth muscle bundles and cells in the circular and longitudinal muscle layers. At the myenteric plexus, telocytes form a complex network enveloping the ganglia (*arrow*) and the nerve strands in the interganglionic region. Telocyte processes appear intermingled with ganglion cells. (D–F) SSc gastric wall. Telocytes are not present in the fibrotic areas of muscle layers. The network of telocytes is discontinuous or even almost completely absent around myenteric plexus ganglia (*arrow*) and nerve strands. CM: circular muscle layer; LM: longitudinal muscle layer; MP: myenteric plexus. Scale bars are indicated in each panel.

In control gastric wall, telocytes were numerous in the muscularis mucosae and submucosa, where they formed a three-dimensional network surrounding vessels and ganglia and an almost continuous layer at the submucosal border of the muscularis propria (Fig. 2A). Conversely, few telocytes entrapped in the fibrotic extracellular matrix were found in the muscularis mucosae and submucosa of SSc gastric wall (Fig. 2B). In the muscularis propria of control sections, telocytes were broadly distributed within the oblique, circular and longitudinal muscle layers and in the myenteric plexus region (Fig. 2C and E). The intramuscular telocytes displayed a small roundish or oval cell body and three or four varicose processes running in every direction around the smooth muscle cells (Fig. 2C and inset). Moreover, telocytes formed a network enveloping the ganglia of the myenteric plexus and were also present along the nerve strands of the interganglionic region (Fig. 2E). In SSc gastric wall, few or no telocytes could be observed in fibrotic areas of muscle layers and around myenteric plexus ganglia (Fig. 2D and F). The findings obtained by using indirect immunoperoxidase methodology were confirmed by immunofluorescence analysis. As shown in Figure 3, in SSc gastric wall, the intramuscular telocytes were severely reduced in the fibrotic areas of muscle layers, and the network of telocytes was discontinuous or even almost completely absent around myenteric plexus ganglia and nerve strands compared with the control specimens. On the contrary, the distribution of telocytes was similar to controls in the healthy-looking areas of muscularis propria (data not shown).

**Fig 4:**
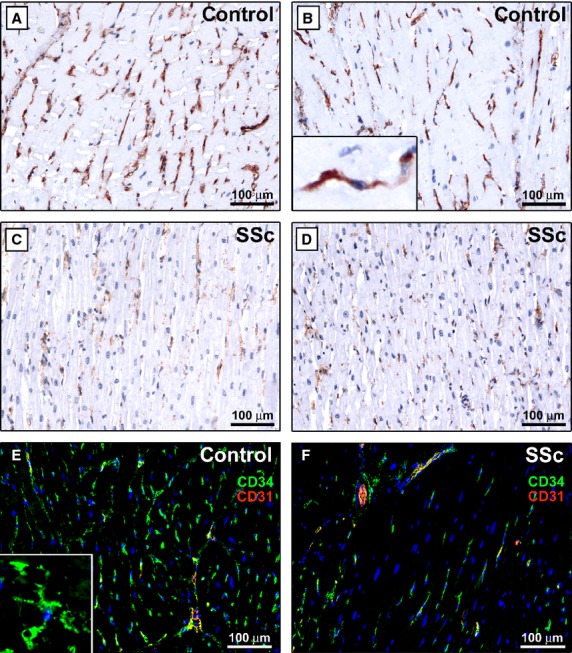
LV myocardium specimens from controls (A, B and E) and patients with systemic sclerosis (SSc) (C, D and F). (A–D) CD34 immunoperoxidase labelling with haematoxylin counterstain. (E and F) Double immunofluorescence labelling for CD34 (green) and CD31 (red) with DAPI (blue) counterstain for nuclei. (A and B) In control myocardium, numerous telocytes are located in the interstitium surrounding the cardiomyocytes. *Inset*: At higher magnification view, a myocardial telocyte displays a small fusiform cell body with two long processes placed between cardiomyocytes. (C and D) In the fibrotic areas of SSc myocardium, telocytes are almost completely undetectable. (E and F) Myocardial telocytes are CD34-positive and CD31-negative, while vascular endothelial cells are CD34/CD31-double-positive. *Inset*: Higher magnification view of a myocardial telocyte with three cellular extensions. Scale bars are indicated in each panel.

In control myocardium, telocytes were numerous and typically located in the interstitium surrounding the cardiomyocytes with a network-like distribution (Fig. 4A, B and E). At higher magnification view, myocardial telocytes displayed a small fusiform cell body with typically two or even three long cytoplasmic prolongations placed between cardiomyocytes (Fig. 4B and E insets), confirming the literature description 31. Conversely, in the fibrotic areas of SSc myocardium, telocytes were severely reduced or almost completely undetectable (Fig. 4C, D and F).

**Fig 5:**
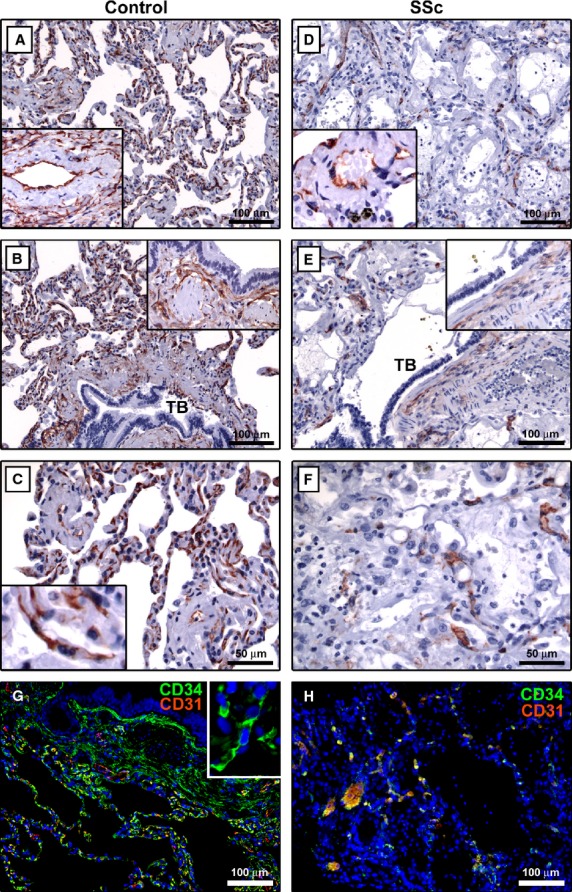
Lung specimens from controls (A–C and G) and patients with systemic sclerosis (SSc) (D–F and H). (A–F) CD34 immunoperoxidase labelling with haematoxylin counterstain. (G and H) Double immunofluorescence labelling for CD34 (green) and CD31 (red) with DAPI (blue) counterstain for nuclei. (A–C) In control lung parenchyma, telocytes are broadly distributed in the interstitial space around alveoli and bronchioles. Telocytes also encircle blood vessels (*inset* in A) and are located around smooth muscle bundles within the wall of bronchioles (*inset* in B). At higher magnification view, lung telocytes display a slender nucleated body and two long varicose processes extending in the pulmonary interstitium (*inset* in C). (D–F) In the fibrotic lung of SSc patients, very few or no telocytes are observed in the thickened alveolar septa and in the interstitial space surrounding terminal and respiratory bronchioles, around blood vessels (*inset* in D) and smooth muscle bundles within the wall of bronchioles (*inset* in E). (G and H) Pulmonary telocytes are CD34-positive and CD31-negative, while vascular endothelial cells are CD34/CD31-double-positive. *Inset*: Higher magnification view of a telocyte with two long cellular extensions. TB: terminal bronchiole. Scale bars are indicated in each panel.

In control lung parenchyma, telocytes were distributed in the interstitial space around alveoli and bronchioles and encircled blood vessels (Fig. 5A–C, G and insets). Moreover, some telocytes were also located around smooth muscle bundles within the wall of bronchioles (Fig. 5B and inset). Telocytes typically exhibited a slender nucleated body and two long, thin and varicose processes extending in the pulmonary interstitium (Fig. 5C and G insets). In the fibrotic lung of SSc patients, very few or no telocytes were found in the thickened alveolar septa, around blood vessels and in the interstitial space surrounding terminal and respiratory bronchioles (Fig. 5D–F, H and insets).

In agreement with previous reports 26,27,30, in all organs, telocytes were CD34-positive and CD31-negative, and therefore they could be clearly distinguished from CD34/CD31-double-positive vascular endothelial cells (Figs 3, 4E, F and 5G, H).

**Fig 6:**
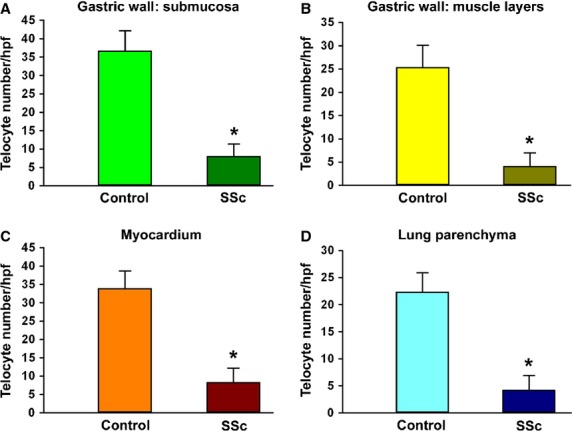
Quantitative analysis of telocytes in sections of gastric wall (A, submucosa; B, muscle layers), myocardium (C) and pulmonary parenchyma (D) from controls and systemic sclerosis (SSc) patients. Data are represented as mean ± SD telocyte number per high-power field (hpf). *P < 0.05 *versus* control (by Student's *t*-test).

As shown in Figure 6, quantitative analysis demonstrated a significant reduction in the number of telocytes in sections from SSc organs compared with the respective controls.

## Discussion

In this study, we investigated for the first time the presence and distribution of telocytes in the internal organs of patients with SSc, a prototypic multisystem fibrotic disorder. Recently, we have shown that telocytes are severely damaged and progressively disappear from the clinically affected skin of SSc patients 27. Herein, we extend our previous findings and clearly show that in SSc, the loss of telocytes is not restricted to the skin, but it is a widespread process affecting multiple visceral organs targeted by the fibrotic process, such as the gastric wall, the myocardium and the lung.

There is firm evidence that, during both development and repair/renewal of tissues and organs, the stromal compartment plays a major role not only by providing support and protection to parenchymal cells but also as a key regulator of tissue homoeostasis, being involved in cell proliferation, survival, differentiation and metabolism 34,35. Fibrosis is a condition characterized by profound changes in the stromal compartment leading to progressive destruction of the normal organ architecture and the consequent impairment of organ function 36. Accordingly, stromal cells, and mainly fibroblasts and myofibroblasts, are considered the principal effector cells involved in the pathophysiology of fibrotic disorders 36–3^38^.

Stromal cells bearing very long cellular extensions have been described in a variety of developing and adult organs, although they have been long neglected and simplistically labelled as fibroblasts. However, in recent years, this view has rapidly changed because of the identification of a peculiar stromal cell type, the telocyte (telos, *i.e*. provided with long-distance cell projections), that appears definitely distinct from the classical fibroblasts 14–3^17^,^23^,^39^. The peculiar ultrastructural phenotype is currently considered as the most reliable hallmark for these cells, which do not possess a unique antigenic profile 14,16. Nevertheless, at present, CD34, a marker shared with vascular endothelial cells, seems the best available choice for the immunohistochemical identification of telocytes under light microscopy 16. In fact, CD34 expression has been firmly reported in telocytes from different organs 16,20,25–3^27^,^29^–3^33^, and by immunoelectron microscopy, it has been demonstrated that the CD34-positive interstitial cells are ultrastructurally identifiable as telocytes 29. Conversely, other markers resulted in a weakly and inconstantly positive immunostaining of telocytes 16,31. Therefore, in the present study, we used CD34 as marker for telocytes. Unfortunately, we could not investigate the ultrastructural features of telocytes by transmission electron microscopy because of the lack of appropriate samples. Indeed, the main limitation to performing such studies is the poor availability of internal organ samples from SSc patients. Therefore, the current study was entirely carried out on archival paraffin-embedded specimens suitable only for light microscopy.

According to the functions that have been proposed for telocytes in different organs 16, their systemic loss might have important pathophysiological implications in SSc. It has been suggested that by their long cytoplasmic processes, telocytes might act as supporting cells and form a scaffold to guide the migration of other cells and the correct assembly of the extracellular matrix, thus contributing to define the correct spatial organization of tissues and organs 16,18,40,41. Therefore, as we have previously proposed for SSc skin 27, the loss of telocytes could mechanically contribute to the altered three-dimensional organization of the extracellular matrix within the stromal compartment of any organ undergoing fibrotic remodelling, such as the gastric wall, the myocardium and the lung. During the fibrotic process, the loss of telocytes might even favour the uncontrolled activation of fibroblasts and their transition to myofibroblasts. Indeed, it has been suggested that telocytes are involved in intercellular signalling, either directly by cell-to-cell contacts, or indirectly by shedding microvesicles and exosomes or secreting paracrine signalling molecules, including microRNAs 16,17,22,24,25,33,42. Interestingly, intercellular contacts between telocytes and fibroblasts or myofibroblasts have been described 25,27,43,44, and thus telocytes could be involved in the maintenance of tissue/organ homoeostasis by controlling fibroblast/myofibroblast activity. In SSc organs, this control is probably impaired because of the reduction and loss of telocytes.

In some organs, such as the heart and the lung, it may be hypothesized that the disappearance of telocytes might impair stem cell-mediated tissue regeneration. Indeed, there is substantial evidence that telocytes might cooperate with stem niches to form tandem cell structures implicated in tissue regeneration and/or repair 15,17,25,33. Moreover, in the myocardium, it has been shown that telocytes and cardiomyocytes are directly connected and might represent a ‘functional unit’, possibly mediating the electrical coupling of cardiomyocytes 31,45. Therefore, it is possible that in SSc, the loss of myocardial telocytes might be implicated in the arrhythmogenesis and disturbances of the cardiac conduction system 5,6,31.

Finally, in the gastrointestinal tract, it has been suggested that telocytes might play a role in the regulation of neurotransmission, possibly contributing to spread the slow waves generated by the ICC 16,26. In SSc, evidence of dysmotility at different levels of the gastrointestinal tract has been reported 7 and has been related to a severe damage of the myenteric neural structures and a reduction in the ICC population 9,10,46. In this context, our findings suggest that in SSc, the loss of the telocyte network in the muscularis propria and myenteric plexus might contribute to gastric dysmotility, particularly delayed gastric emptying or gastroparesis 7.

In conclusion, together with previous findings 27,30,40, the data reported in this study clearly point to a broad involvement of telocytes in the fibrotic remodelling of multiple organs in SSc and other conditions characterized by fibrosis. Indeed, in a recent study, telocytes were found to be decreased during experimental myocardial infarction, particularly in fibrotic areas, and the transplantation of cardiac telocytes in the infarcted and border zones of the heart decreased the infarction size and improved myocardial function 40. Moreover, our group has recently demonstrated that a loss of telocytes accompanies the fibrotic remodelling of the intestinal wall in patients affected by Crohn's disease 30. In SSc and other fibrotic conditions, the loss of telocytes might become, in the near future, matter for an early targeted therapy to control the evolution to irreversible tissue fibrosis. For this reason, further functional studies are necessary to identify the pathogenetic mechanisms involved in telocyte loss and the role of these stromal cells in the regulation of the fibrotic process.
